# The role of R-loops-mediated epigenetic regulation in genome stability maintenance and disease pathogenesis: a systematic review

**DOI:** 10.1080/15592294.2026.2653959

**Published:** 2026-04-13

**Authors:** Zi Hao Jiang, Di Feng Wang, Rui Shao, Xing Sheng Wang, Zi Ren Tang

**Affiliations:** a Department of Emergency Medicine, Beijing Chaoyang Hospital, Beijing Key Laboratory of Cardiopulmonary-Cerebral Resuscitation Innovation and Translation, Capital Medical University, Beijing, China; b Department Obstetrics and Gynecology, Zhuji Maternity and Child Health Hospital, Zhuji, China

**Keywords:** R-loops, epigenetic regulation, human diseases, molecular mechanisms

## Abstract

This systematic review synthesizes current evidence linking R-loops – associated processes with epigenetic regulation, genome stability, and human disease, integrating findings across DNA/RNA/protein modifications and 3D genome organization, and highlighting key mechanistic and technical gaps relevant to future studies, including longer-term translational exploration. Following PRISMA guidelines (Registration No.: INPLASY202560055), PubMed and ScienceDirect databases were searched through 18 January 2026. Two independent reviewers conducted blinded screening via the Rayyan platform. From 1290 initial articles, 71 studies met inclusion criteria. Methodological rigour was assessed using a modified SYRCLE risk-of-bias tool. Thematic synthesis identifies R-loops as recurrent intermediates linking transcription, chromatin regulation, and genome maintenance; feedback-like relationships are reported in selected systems. They spatially impede DNMTs to sustain promoter hypomethylation while recruiting TET enzymes for demethylation, and RNA modifications (e.g. m5C/m6A) may tune hybrid stability and, in some contexts, influence effector recruitment. R-loops facilitate the recruitment of histone-modifying complexes (PRC1/PRC2, G9a/GLP), thereby influencing chromatin landscapes and 3D genome architecture – including associations with TAD boundary insulation and with LLPS-related repair compartments in specific contexts. Dysregulation of R-loop homeostasis links to disease-related epigenetic alterations and genome instability with distinct disease-, locus- and cell-state specificity, and its context-dependent roles in diverse disorders involve transcriptional silencing, DNA damage, replication-transcription conflicts, repair pathways, chromatin regulation and mitochondrial genome maintenance. Integrating DRIP-seq with epigenomic profiling and functional tests supports mapping of context-specific relationships.

## GRAPHICAL ABSTRACT

**Figure:**
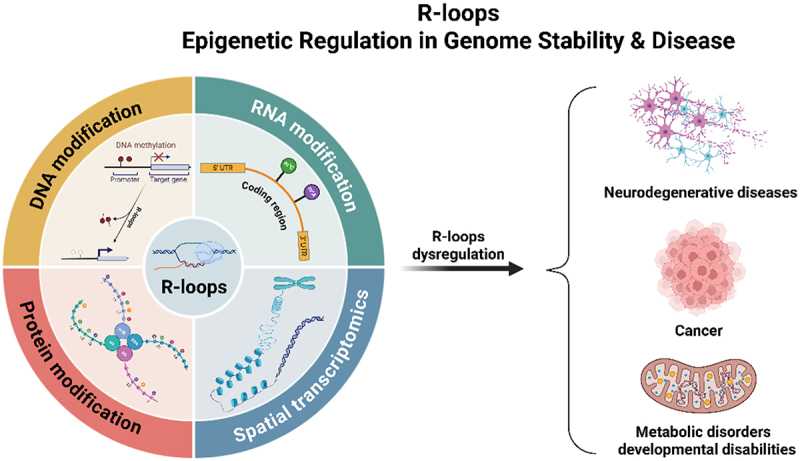

## Introduction

Epigenomics aims to untangle how chemical modifications and spatial conformational changes of the genome regulate gene function and expression without altering the nucleotide sequence [1–3]. In recent years, with the rapid advancement of omics and high-resolution spatial technologies, R-loops – a three-stranded nucleic acid structure formed by the hybridization of nascent RNA with its DNA template [4,5] —have garnered increasing attention for their pivotal roles in genomic stability and epigenetic regulation [6,7]. Within R-loops, the RNA strand forms a hybrid duplex with the template DNA strand, displacing the non-template strand and creating a distinctive single-stranded DNA (ssDNA) region [8]. Originally considered passive byproducts of transcription, R-loops are currently regarded as essential molecular intermediaries that integrate genetic and epigenetic mechanisms of gene control⁠ [9].

Emerging evidence indicates that R-loops extend beyond their roles in transcription, genome stability, and DNA repair to exert considerable impact on alterations in chromatin conformation, DNA methylation and demethylation processes, as well as a diverse array of post-translational modifications affecting both histone and non-histone proteins [10,11]. Furthermore, R-loops participate in higher-order chromatin folding and spatial genome organization, directly mediating the fine-tuned regulation of gene accessibility and expression profiles [12,13]. Disruptions in R-loops homeostasis are firmly associated with the mechanisms underlying several major human diseases, including cancers, neurodegenerative conditions, and developmental anomalies [14,15]. These findings underscore the importance of elucidating the molecular mechanisms of R-loops for understanding disease initiation and progression, and they may ultimately inform intervention strategies; however, most current evidence remains preclinical and context-dependent, making direct clinical translation premature. Despite accumulating evidence, the integrative role of R-loops within the multi-layered epigenomic remains to be systematically elucidated. Their bridging function between DNA methylation and demethylation, RNA modifications (e.g., m^6^A methylation, pseudouridylation), and various post-translational protein modifications positions them as potential core regulators in the genetic and epigenetic networks [16]. Furthermore, their expanding roles in three-dimensional genome organization, cellular differentiation, and disease pathogenesis provide novel insights into disease mechanisms and may, in the longer term, inform precision medicine efforts once robust and standardized R-loop assays with adequate specificity and reproducibility become available [17].

This review systematically summarizes recent advances in R-loops within the field of epigenomics, with a focus on their molecular origins, regulatory functions, and interaction mechanisms with DNA/RNA/protein modifications as well as spatial genomics. Special emphasis is placed on their potential roles in human health and disease, along with prospective applications. It is anticipated that this synthesis will facilitate mechanistic investigations and help prioritize longer-term translational directions. Nevertheless, therapeutic development will require improved locus- and context-specificity, mitigation of pleiotropic effects, and clinically feasible methods to monitor R-loops dynamics.

## Method

### Study protocol and registration

This systematic review was rigorously designed and executed in adherence to the PRISMA guidelines, which represent the Preferred Reporting Items for Systematic Reviews and Meta-Analyses. The study protocol was formally registered with the International Platform of Registered Systematic Review and Meta-Analysis Protocols, known as INPLASY, and assigned the registration number INPLASY202560055 as well as the digital object identifier https://10.37766/inplasy2025.6.0055.The registration details are publicly accessible on the INPLASY website (https://inplasy.com/).

### Inclusion and exclusion criteria

Eligible studies included original experimental research and high-quality omics analyses investigating R-loops-induced epigenetic modifications and their molecular mechanisms and pathological consequences in human diseases.

### Inclusion criteria

Study Subjects: Human cell lines, tissue samples, and animal models relevant to human diseases, with a particular focus on cancer and neurodegenerative disorders.

Research Content: Studies explicitly reporting the relationship between R-loops formation/dynamic regulation and epigenetic alterations, including DNA methylation, histone modifications, and chromatin remodelling.

Experimental Methods: Utilization of molecular techniques such as DRIP (DNA-RNA immunoprecipitation), ChIP-seq, RNA-seq, and whole-genome methylation sequencing, among others, with validation of R-loops and epigenetic states.

Language: Only English-language publications were included.

Publication Date: No time restrictions were applied to ensure comprehensive retrieval.

### Exclusion Criteria

Studies conducted in non-disease, non-human systems , case reports lacking mechanistic data, reviews, commentaries, and purely computational modelling studies were excluded.

### Data sources and search strategy

A systematic search was performed in the PubMed and Science Direct databases, with the retrieval date set to 18 January 2026.The search strategy incorporated both Medical Subject Headings (MeSH) terms and keywords, such as ‘R-loops,’ ‘epigenetic modification,’ ‘DNA methylation,’ ‘histone modification,’ ‘chromatin remodeling,’ and ‘human diseases.’ The detailed search queries are provided in the Appendix.

### Literature screening and data extraction

During the literature screening process, the Rayyan literature management platform was utilized to assist with the initial screening of titles and abstracts. Two independent reviewers performed blinded evaluations of all retrieved records on the Rayyan platform to enhance screening efficiency and accuracy [18]. Articles meeting the inclusion criteria subsequently underwent full-text review and assessment. In cases of disagreement during the screening process, unresolved discrepancies were resolved through consultation with a third-party expert. Data extraction was conducted using a pre-designed standardized form, which included basic study information, disease model types, R-loops detection and epigenetic modification methods, key molecular mechanisms, pathological impacts, and core experimental findings. The data extraction process was independently performed by two reviewers, with the Rayyan platform facilitating literature management and tracking screening progress to ensure procedural standardization and traceability.

### Quality assessment

Risk of bias was assessed using a modified SYRCLE tool adapted for mechanistic R-loop/epigenetics studies, where randomization/blinding are often incompletely reported and assay validity can be a major determinant of inference. Each included study was evaluated across 10 prespecified domains: (1) sequence generation (randomization), (2) allocation concealment, (3) blinding of implementation, (4) blinding of outcome assessment, (5) data integrity/completeness, (6) selective reporting, (7) experimental reproducibility, (8) antibody/reagent specificity, (9) model/sample confirmation, and (10) statistical methods (Supplementary Table S2). Overall risk of bias per study was categorized using predefined thresholds: low risk if >70% of applicable items were rated low risk, high risk if >30% of applicable items were rated high risk, and unclear risk otherwise. Two reviewers independently assessed risk of bias; disagreements were resolved by discussion and, if needed, adjudication by a third reviewer.

### Data synthesis

Given the heterogeneity in study designs and outcomes, this review employed a combined approach of qualitative description and thematic synthesis to summarize the molecular mechanisms of R-loops-mediated epigenetic remodelling and its pathological consequences. To avoid over-interpreting associative datasets, we additionally annotated the strength of inference for 3D genome/LLPS-related statements as: (i) correlative co-localization, (ii) functional perturbation, or (iii) mechanistic/causal evidence (reconstitution and/or perturbation – rescue with direct readouts).

## Result

**Figure 1.**
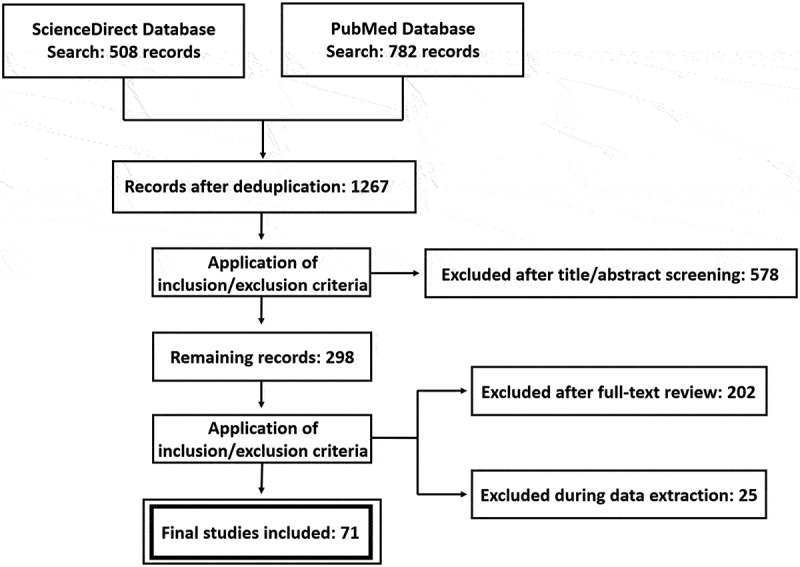
PRISMA flow diagram of literature screening.

The initial literature search identified 1290 potentially relevant articles from the PubMed and ScienceDirect databases, with 782 records originating from PubMed and 508 from ScienceDirect. After removing duplicates and reviewing titles and abstracts, publications that clearly failed to meet the inclusion criteria were excluded, resulting in 298 articles retained for full-text assessment. Finally, applying the predefined inclusion and exclusion criteria, 71 studies were deemed eligible and included as the analytical dataset for this systematic review. The complete literature selection process is illustrated in the PRISMA flow diagram (Figure 1).

### Risk of bias across included studies

Risk of bias was assessed for all included studies (*n* = 71; literature search updated through 18 January 2026) using an adapted SYRCLE-based tool tailored to mechanistic R-loop/epigenetics research (10 domains; Supplementary Table S2). The most frequent concerns were lack of blinding of outcome assessment (high risk: 29/71) and incomplete confirmation/characterization of experimental models or biological samples (model/sample confirmation: high risk 20/71; unclear 13/71). Uncertainty regarding antibody/reagent specificity and/or limited reporting of orthogonal validation for R-loop detection was also common (unclear: 14/71). Selective reporting was judged unclear in 11/71 studies, most often because key negative controls and/or null findings were not consistently reported when interpreting ‘R-loop stabilization/increase’ (e.g., incomplete documentation of RNase H sensitivity where applicable, reciprocal perturbations, or complementary validation). By contrast, data completeness was consistently low risk (71/71), and relatively few studies were judged unclear regarding statistical methods (4/71) or experiment reproducibility (unclear 4/71; high 1/71). Full study-level ratings and operational decision rules are provided in Supplementary Table S2.

**RQ1**: How do R-loops and epigenetic chemical modifications (e.g., DNA methylation, RNA modifications, histone modifications) influence each other in a locus- and context-dependent manner, and what evidence supports mechanistic coupling relevant to gene expression and genome stability?

### Interaction mechanisms between R-loops and DNA methylation, hydroxymethylation, and demethylation.

#### Mechanistic and correlative links between R-loops and DNA methylation dynamics at selected loci

**Figure 2.**
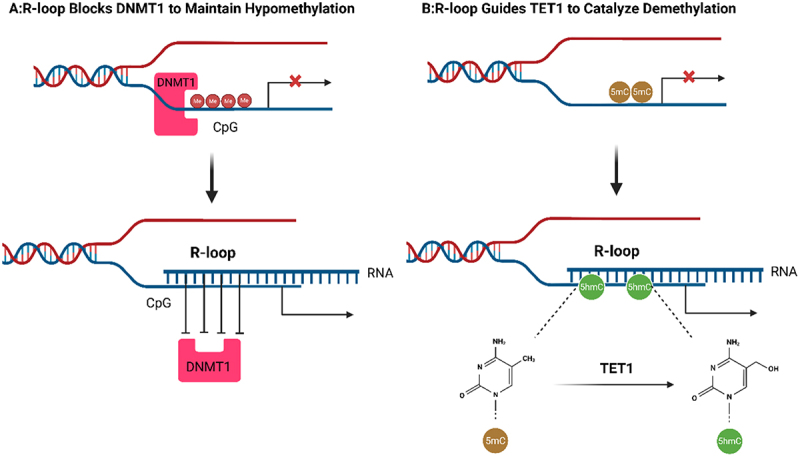
R-loops modulate DNA methylation status. This figure illustrates the functional role of R-loops in the dynamic regulation of DNA methylation. In pathway A, R-loops physically obstruct the binding of DNMT1 to CpG sites in promoter regions through steric hindrance, thereby maintaining the hypomethylated state of CpG islands and activating gene transcription. The pathway B demonstrates that R-loops serve as molecular scaffolds mediating the targeted recruitment of TET1, which catalyzes the oxidation of 5-methylcytosine (5mC) to 5-hydroxymethylcytosine (5hmC), consistent with active DNA demethylation in those locus- and system-specific settings. Schematic summarizes models supported in selected loci/systems; broader generality, directionality, and integration across pathways may vary by context. (created with Biorender.com).

Although multiple studies have demonstrated spatial correlations between R-loops and DNA methylation patterns, direct mechanistic evidence remains limited to a few loci such as SEMA3F and TCF21.R-loops have been reported to correlate with DNA methylation dynamics through dual pathways involving physical obstruction and enzyme recruitment. Functioning as spatial barriers, R-loops impede the binding of DNMT1/DNMT3b to promoter regions of genes such as SEMA3F and SNRPN, thereby maintaining CpG island hypomethylation to facilitate transcriptional activation [19–21]. Concurrently, R-loops may facilitate the recruitment of TET1/3 by GADD45A or DHX33 to loci such as DSP and TCF21, catalysing the oxidation of 5mC to 5hmC to facilitate active demethylation [22–24]. In addition, recent evidence links the active demethylation machinery more directly to RNA biology and RNA:DNA hybrids, supporting a mechanistic coupling between TET‑associated demethylation pathways and R-loop contexts [25]. These dual mechanisms through which R-loops modulate DNA methylation status are summarized in Figure 2. The resulting 5hmC enhances DNA breathing dynamics, reducing the stability of the DNA double helix and significantly improving the annealing efficiency between the template strand and nascent RNA, thereby facilitating R-loop formation. In specific experimental systems, these observations have been interpreted as a potential reciprocal reinforcement between local 5hmC accumulation and R-loop propensity, where R-loops favour 5hmC generation and 5hmC may in turn facilitate hybrid formation [26,27]. However, direct evidence for a generalized positive feedback loop across loci remains limited, and the extent of reciprocity is likely to depend on chromatin context and the availability of relevant methylation, demethylation, and hybrid-resolving factors.

#### Potential influence of DNA methylation status on R-loops stability

DNA methylation and hydroxymethylation inversely modulate R-loop stability through structural remodelling: CpG methylation (5mC) induces the formation of Z-type DNA-RNA hybrids, which impede DNA polymerase binding by narrowing the minor groove and reducing helical diameter, ultimately leading to replication fork stalling [27,28]. In contrast, TET enzyme-mediated 5hmC decreases DNA duplex stability, significantly enhancing the annealing efficiency between the template strand and nascent RNA, thereby promoting R-loops enrichment at transcription termination sites and modulating differentiation-related pathways in embryonic stem cells, such as mTOR [26]. Perturbation of DNMT/TET activity and hybrid homeostasis has been implicated in disease-associated epigenetic states and genome instability in a context-dependent manner: in prostate cancer, a proposed R-loop – m6A – IGF2BPs axis has been reported to be associated with tumour suppressor genes by blocking DNMT1 [19,29], whereas loss of TET2/3 results in 5hmC depletion, leading to aberrant accumulation of R-loops and G-quadruplexes (G4s), and has been linked to lymphoma-associated phenotypes [27,29]. These findings collectively support a bidirectional association rather than a universally causal mechanism.

### RNA chemical modifications tune R-loops stability: potential links to genome topology and drug response.

**Figure 3.**
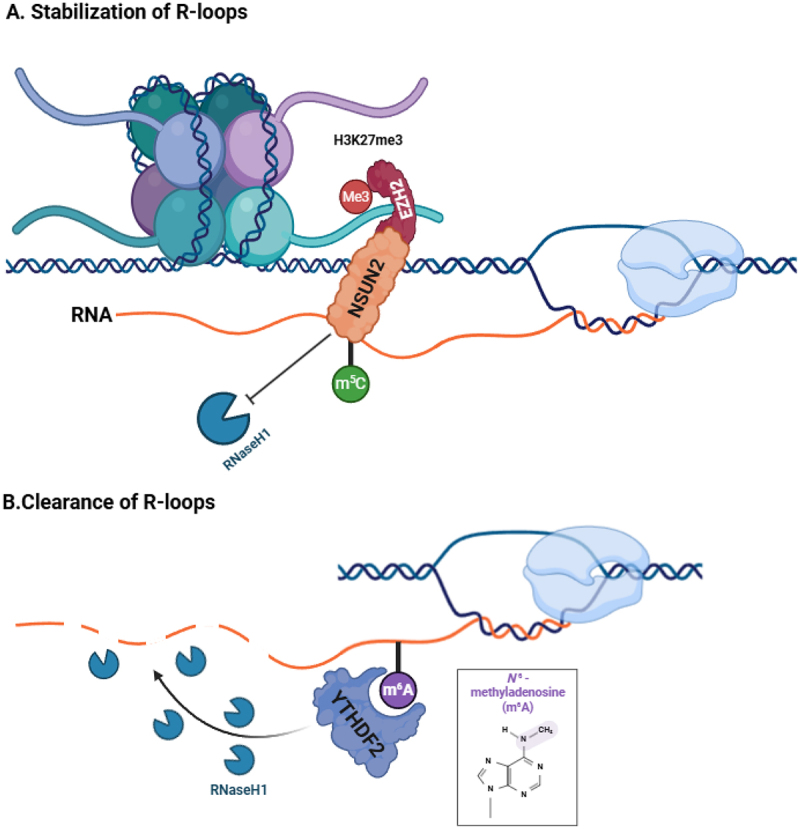
Context-dependent effects of RNA modifications on R-loop stabilization and resolution. A. R-loop stabilization pathway: NSUN2 catalyzes m5C modification, which enhances the stability of RNA – DNA hybrids and directly counteracts their degradation by RNase H1, thereby prolonging the half-life of R-loops. Subsequently, components such as EZH2 are recruited to catalyze the deposition of H3K27me3 at tumor suppressor gene loci, promoting localized chromatin epigenetic silencing. B. R-loop resolution pathway: YTHDF2 specifically recognizes m6A-modified R-loops and collaborates with RNase H1 for targeted degradation. Schematic summarizes models supported in selected loci/systems; broader generality, directionality, and integration across pathways may vary by context. (created with Biorender.com).

Emerging studies reveal both correlative and mechanistic interactions between RNA modifications and R-loops stability. Available evidence suggests that RNA epigenetic marks can modulate R-loops stability in opposite directions depending on locus, cell state, and the specific reader/effector proteins engaged (Fig 3): Stabilization Effect: NSUN2-mediated m^5^C modification has been shown to associate with enhanced R-loop stability and directly counteracts their degradation by RNase H1, thereby prolonging the half-life of R-loops. This subsequently recruits EZH2 to tumour suppressor gene loci, including PRDM11, catalysing the deposition of H3K27me3 and promoting epigenetic silencing [30]. Similarly, METTL3-mediated m^6^A modification of TERRA promotes the formation and stabilization of R-loops at telomeric regions by recruiting hnRNPA2B1 protein [31]. Promotion of clearance pathways: YTHDF2 recognizes m^6^A-modified R-loops and collaborates with RNaseH1 to degrade them, and its absence leads to failure in DNA damage response [32–35]. TET1-mediated m^5^C demethylation reduces R-loops affinity, facilitating their orderly dissociation during the late stages of DNA repair [36].

This balance has been proposed in specific models, but its generality remains unclear.

During the early phase of DNA damage repair, TRDMT1 deposits m^5^C to enhance the binding affinity of RAD52 to R-loops [37], while in the later phase, TET1 erases m^5^C to facilitate their resolution [36], suggesting a sequential model in which distinct RNA-modification enzymes may act at different stages of repair. However, evidence for a generalizable circuit is limited, and disease relevance is currently supported mainly in preclinical, context-specific systems. In glioblastoma, reduced nuclear export of m^6^A-modified circPOLR2B leads to increased intra-nuclear R-loops formation, which alleviates transcriptional repression of the host gene POLR2B and activates the downstream oncogenic PBX1 signalling pathway [38]. In bladder cancer patients, NSUN2 overexpression demonstrates positive correlation with R-loop accumulation [30]. Several studies provide preclinical proof-of-concept that perturbing RNA-modifying enzymes can alter R-loops homeostasis in a context-dependent manner. For instance, in an HCMV infection model, the METTL3 inhibitor STM2457 was reported to reduce m^6^A-associated aberrant R-loops accumulation [39]. However, given the broad roles of METTL3 in RNA metabolism, whether disease-relevant phenotypes are mediated specifically through R-loops modulation remains uncertain. Similarly, NSUN2 suppression (e.g., siRNA) in bladder cancer models was associated with altered R-loops stability and increased cisplatin sensitivity [30], but further work is needed to disentangle direct R-loops-dependent mechanisms from pleiotropic transcriptional effects. We refer to R-loops as a likely mechanistic mediator only when hybrid perturbation/rescue links RNA marks to phenotype; otherwise RNA modifications may affect hybrids indirectly via transcript processing. CRISPR alone is insufficient; add direct R-loop readouts and RNaseH1 rescue (catalytic controls). Future research should also address the spatial specificity paradox – why the same m^6^A mark may stabilize telomeric R-loops via hnRNPA2B1 while promoting clearance at promoters in an ARID1A-dependent manner. Finally, although single-cell sequencing highlights tumour-subtype dependencies on specific RNA-modification pathways (e.g., METTL8–m^3^C in glioma stem cells) [40], translating such observations into patient stratification remains a longer-term goal; at present, the lack of standardized, high-specificity and clinically scalable R-loop assays, together with incomplete causal attribution, makes clinical implementation premature. Progress will require assay harmonization, orthogonal validation, and prospective cohort studies. Overall, RNA chemical modifications may influence genome structural remodelling through dynamic regulation of R-loops stability, but their contributions to tumour epigenetic dysregulation are likely to be locus- and context-dependent.

### Multi-level post-translational modification crosstalk in R-loops – prone contexts and genome homeostasis.

#### R-loops regulate histone modifications by remodeling the local chromatin environment

**Figure 4.**
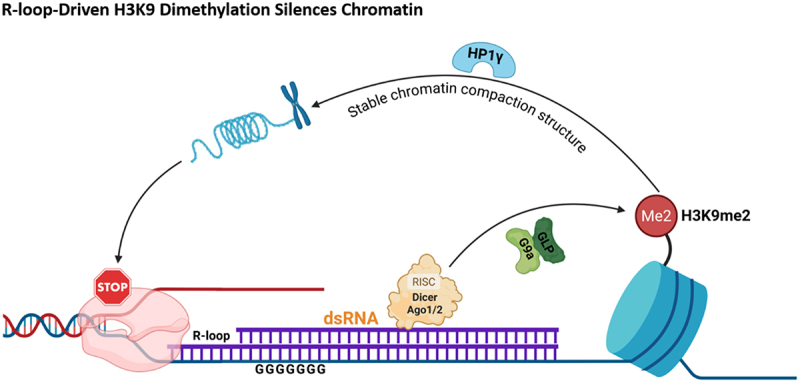
R-loops mediate histone methylation to stabilize chromatin compaction for transcription termination. R-loops facilitate the generation of double-stranded RNA (dsRNA), thereby recruiting the RNA-induced silencing complex (RISC) containing dicer and AGO1/2. R-loops have been reported to facilitate dsRNA generation and recruitment of RNAi machinery (dicer/AGO), which can in turn recruit G9a/GLP and promote local H3K9me2 and HP1γ enrichment, associated with pol ii pausing at termination regions. Schematic summarizes models supported in selected loci/systems; broader generality, directionality, and integration across pathways may vary by context. (created with BioRender.com).

The RNA – DNA hybrid structure of R-loops can serve as a potential binding platform that facilitates recruitment or stabilization of epigenetic complexes at specific genomic loci. For instance, within the gene termination region, such as at the β-actin locus, R-loop formation can promote downstream antisense transcription, leading to the production of double-stranded RNA. This dsRNA can recruit components of the RNA interference pathway, including Dicer and Argonaute 2 (Ago2), as well as the histone methyltransferase G9a/GLP complex. The G9a/GLP complex catalyses the deposition of dimethylation at lysine 9 of histone H3 (H3K9me2) and recruits heterochromatin protein 1 gamma (HP1γ), forming a repressive chromatin domain. This process is associated with RNA polymerase II pausing and participates in the regulation of transcription termination [41]. This mechanistic pathway, linking R-loops to chromatin compaction via histone methylation, is visually summarized in Figure 4. At PRC-regulated genes, including developmental loci such as HOX clusters, R-loop accumulation has been associated with increased chromatin retention of RING1B and with H2AK119ub1/H3K27me3 at a subset of loci; whether this reflects a direct cascade is context-dependent. In these systems, the Polycomb-marked state is consistent with transcriptional silencing of target genes [42]. However, the extent to which they reflect a unified, hybrid-driven recruitment cascade versus parallel chromatin features may vary across loci and remains to be clarified. In the nucleolar region, pathological conditions such as SETX deficiency lead to abnormal accumulation of R-loops, which significantly reduces H3K9ac/H4K16ac levels, disrupts open chromatin structure, interferes with rRNA processing, and ultimately triggers nucleolar dysfunction [43–45].

#### Ubiquitination pathways linking R-loop – prone contexts to DNA repair factor stability and localization

Multiple studies indicate that ubiquitination and deubiquitination can influence the stability, activity, and localization of DNA repair factors in R-loop – prone contexts. Although these events are often characterized in separate experimental systems and may not constitute a single hierarchical pathway, together they suggest a regulatory linkage between hybrid-associated transcription/replication stress and repair capacity.Regulation of Repair Factor Stability:USP11 counteracts KEAP1-mediated K48-linked polyubiquitination of SETX, thereby stabilizing SETX and preserving its ability to resolve R-loops [46]. Similarly, TOP3B stability is maintained through a balance between degradation and deubiquitination: MIB1 promotes K11/K48-linked ubiquitination and proteasomal turnover of TOP3B, whereas the TDRD3–USP9X complex removes these ubiquitin chains to stabilize TOP3B. This dynamic equilibrium prevents the accumulation of toxic TOP3B cleavage complexes and safeguards R-loop homeostasis [47].

Regulation of Repair Factor Localization and Function: Ubiquitination also determines the spatial deployment of repair factors. RNF8-mediated ubiquitination of XRN2 facilitates its recruitment to R-loop – prone regions, such as transcription termination sites, enabling efficient R-loop resolution [48]. Under replication stress, RAD18 catalyses monoubiquitination of PCNA at K164, promoting the recruitment of the Fanconi anaemia core protein FANCD2 to stalled replication forks and enhancing homologous recombination repair [49].

Chromatin Environment and Contextual Regulation: Beyond individual proteins, ubiquitin signalling modulates the chromatin environment surrounding R-loops. Mdm2 and RNF2 catalyse monoubiquitination of histone H2A at lysine 119 (H2AK119ub), reinforcing a repressive chromatin state that limits aberrant transcription-replication conflicts. Reduced H2AK119ub levels correlate with R-loop accumulation and replication fork dysfunction [50,51]. Collectively, these findings support that ubiquitin signalling can modulate repair factor deployment and local chromatin environments in settings where RNA:DNA hybrids accumulate. The extent to which these ubiquitin-dependent processes form a coordinated network across contexts remains to be established.

#### Crosstalk between R-loops and phosphorylation, acetylation, ADP-ribosylation, and phase separation in genome stability contexts

Across studies, phosphorylation, acetylation, ADP-ribosylation, and phase separation have been linked to R-loop formation or resolution, suggesting multiple points of crosstalk. However, directionality and integration of these links are context-dependent and do not yet support a single unified regulatory network. Specifically, Ser2/Ser5 phosphorylation of the RNA polymerase II carboxyl-terminal domain (CTD) modulates transcription elongation efficiency, thereby indirectly influencing R-loops formation propensity [52]. Acetylation modification exhibits a dual regulatory effect – SIRT7-catalysed deacetylation of DDX21 enhances R-loop clearance capacity [53], whereas excessive elevation of histone H3K9ac induced by HDAC inhibitors leads to alterations in chromatin structure and increases the risk of R-loop accumulation [29]. At the DNA Damage Response Level: Under SRSF2-mutant conditions, R-loops activate PARP1-catalysed PARylation modifications, recruiting RNase H1/H2 to initiate repair [54]. Conversely, ADP-ribosylation of HMGA1 maintains replication fork stability through NUDT16-driven reversal modifications, independently mediating chemotherapy resistance [55]. Higher-order regulation is achieved through phase separation, wherein telomeric repeat-containing RNA (TERRA) forms liquid-liquid phase separation condensates with LSD1 via its G-quadruplex structure, promoting the formation of local R-loops [56].

### The pivotal role of R-loops in 3D genome remodeling: from spatial barriers to epigenetic memory programming

#### R-loop-mediated remodeling of 3D genome spatial architecture

**Figure 5.**
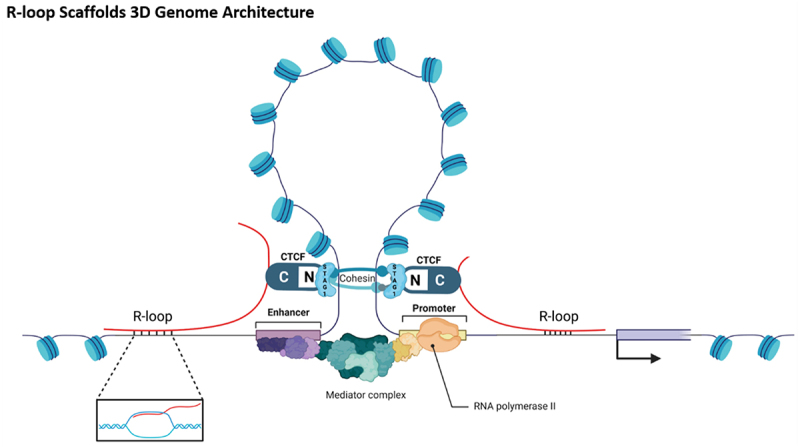
CTCF orientation-dependent chromatin looping and R-loop-mediated spatial gene Regulation. CTCF protein binds to the STAG1 subunit of the cohesin complex via its N-terminal domain, establishing a directional barrier at specific genomic loci to block DNA loop extrusion. Concurrently, R-loops are enriched at some boundary-like regions and, in selected loci, may reinforce boundary activity and associated enhancer – promoter proximity. However, genome-wide co-localization between R-loop maps and architectural features is often correlative; causal inference typically requires perturbation/rescue (e.g., targeted RNase H or CRISPR-based boundary perturbations) coupled with direct 3D readouts (hi-C/Micro-C/3C or imaging). Mediator is shown schematically as a canonical coactivator. (created with Biorender.com).

R-loops have been linked to changes in higher-order chromatin organization in specific contexts, with evidence ranging from locus-level mechanistic studies (including reconstitution) to broader correlative overlaps with 3D genome features (Figure 5).R-loops and cohesin-mediated loop extrusion frequently meet at boundary-like regions. Building on this colocalization, it has been proposed that steric or topological effects of hybrids may hinder loop extrusion and thereby help define TAD boundaries in specific settings.Consistent with this model, single-molecule reconstitution showed that RNA:DNA hybrids can directly stall cohesin-mediated DNA compaction in vitro: cohesin stalled ~19 ± 8 kb after its first collision with an R-loop and required ~5 ± 2 collisions on average for complete arrest. In contrast, genome-wide overlap between R-loop maps and insulation/TAD features is often associative and may be confounded by transcription/RNAPII-linked insulation unless supported by perturbation – rescue designs [12]. Such interference may contribute to insulation at boundary-like regions in specific settings, but genome-wide inference is often correlative [12,13]. Secondly, at telomeric regions, the specifically expressed TERRA long non-coding RNA can facilitate the formation of G-quadruplex DNA through R-loop formation. This R-loops to G-quadruplex conformational transition is crucial for telomere homeostasis maintenance and is profoundly associated with the activation mechanism of the alternative lengthening of telomeres (ALT) pathway [57]. Thirdly, in DNA damage contexts, LLPS-based repair compartments can be R-loops-dependent, and R-loop structures may contribute to nucleic-acid scaffolding within these condensates. During this process, the phosphorylated SOSS1 complex dynamically assembles membrane-less repair compartments at DNA double-strand break (DSB) sites through synergistic interactions with the phosphorylated CTD domain of RNA polymerase II. The spatially specific enrichment effect of these compartments efficiently recruits and concentrates DNA damage repair factors, significantly enhancing the spatial coordination of the damage response [58]. Whether R-loops nucleate condensates or are enriched as passengers within pre-existing assemblies remains to be clarified.

#### The coupling mechanism of R-loop-mediated epigenetics and three-dimensional genome architecture

As potential epigenetic – structural coupling interfaces, R-loops influence gene expression by dynamically regulating chromatin spatial organization. In highly transcribed regions, R-loops formation is generally linked to an open chromatin configuration, which can subsequently influence specific histone modifications, including H3K4me3, via changes in local DNA topology [6]. A representative example is observed at oestrogen-responsive loci, where RING1B enhances enhancer-promoter looping by facilitating R-loops formation, ultimately activating the transcription of target genes [59]. Concurrently, R-loops and their associated G-quadruplex (G4) structures have been found to be enriched at CTCF binding pockets and enhance CTCF binding [60]. Notably, during neural development, ADNP deficiency leads to abnormal R-loops accumulation at CTCF sites, disrupting chromatin loop structures and impairing neuronal differentiation [61]. This observation provides a direct mechanistic route by which R-loops dysregulation at architectural elements (CTCF/cohesin) can translate into altered loop/TAD wiring and neurodevelopmental phenotypes, beyond DNA damage alone. Furthermore, RNA derived from Alu repeat elements is predicted to mediate long-range chromatin interactions through the formation of trans-chromosomal R-loops [62]. Although these spatial associations are strongly suggestive, direct causative evidence linking R-loops to global 3D genome remodelling remains limited.

RQ2: How does the interaction between R-loops and epigenetic modifications contribute to disease pathogenesis?

Current evidence supports both correlative associations and functional contributions of R-loops to epigenetic dysregulation in neurodegeneration, cancer, and metabolic/developmental disorders; however, mechanistic causality has been demonstrated only in selected loci and experimental systems. Importantly, the biological consequences of R-loops appear to be highly context-dependent, shaped by (i) disease-specific upstream triggers (e.g., repeat expansions, replication stress, splicing defects, or chromatin-modifier dysfunction), (ii) genomic/chromatin environments (repetitive heterochromatin, CpG-island promoters, telomeres, long genes, or mitochondrial compartments), and (iii) cellular states and vulnerability (post-mitotic neurons, proliferating tumour cells, metabolically stressed hepatocytes, or differentiating stem/progenitor cells). Accordingly, we summarize below disease- and locus-specific mechanisms and highlight where evidence supports causality versus association.

#### Neurodegenerative diseases: disease- and locus-specific epigenetic and DNA damage mechanisms involving R-loops

**Figure 6.**
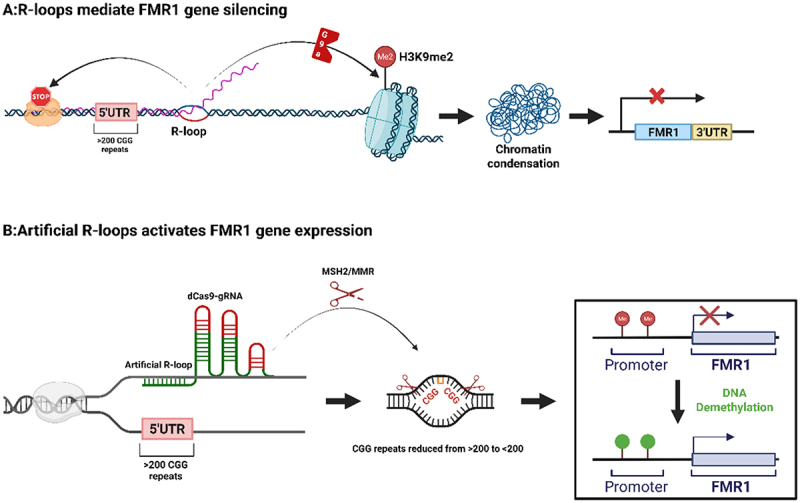
From lock to key: the role reversal of R-loops in fragile X Syndrome. this figure untangles the dual roles of R-loops in modulating the FMR1 gene: panel a demonstrates that natural R-loops form stable triplex structures upon CGG repeat expansion (>200 repeats), recruiting the G9a methyltransferase to deposit H3K9me2, thereby compacting chromatin and stalling RNA polymerase ii, ultimately silencing FMR1 expression. Panel B illustrates that artificial R-loops, induced by the dCas9-gRNA system at the CGG repeat region, recruit the MSH2/MMR repair complex to precisely excise excess repeats (>200→<200), promoting promoter demethylation and restoring FMR1 transcription. (created with Biorender.com).

In neurodegenerative disorders, altered R-loop homeostasis has been linked to disease-relevant epigenetic states and DNA damage responses; however, the dominant upstream triggers, chromatin contexts, and downstream consequences differ substantially among diseases, cell types, and genomic loci.Repeat-expansion – associated chromatin repression. In Friedreich’s ataxia (FRDA), R-loops stably accumulate within the pathogenic GAA repeat expansion region of the FXN gene, directly impeding transcriptional elongation. Furthermore, They are reported to recruit or may facilitate the recruitment of G9a, which catalyses the deposition of H3K9 dimethylation (H3K9me2), thereby promoting heterochromatin formation and FXN gene silencing [63]. This repeat-expansion – linked G9a/GLP – H3K9me2 heterochromatin route is prominent in post-mitotic neurodegenerative contexts, and contrasts with many proliferative cancers where Polycomb-linked chromatin compaction defects and replication-stress landscapes more frequently intersect with RNA:DNA hybrid accumulation (see Cancer section).In fragile X syndrome, R-loops can serve as both pathogenic factors and therapeutic targets. Under pathological conditions, R-loops may also silence the FMR1 gene through the aforementioned mechanisms [63]. Notably, targeted induction of transient R-loops has been used as an experimental proof-of-principle approach to promote repeat excision and gene reactivation in model systems.This approach facilitates CGG repeat excision through recruitment of the MSH2 mismatch repair complex, leading to repeat contraction and subsequent DNA demethylation, thereby achieving sustained reactivation of FMR1 gene expression [64]. (Figure 6). This illustrates that the same structural intermediate may have divergent outcomes depending on how, where, and when it is formed. Consistent with this context dependence, a 2025 study identified an SVA retrotransposon – embedded hexamer repeat RNA that drives R-loops formation and neurodegenerative phenotypes, and knockdown of the repeat-containing RNA by antisense oligonucleotides rescued apoptosis in organoid models [65].

Repeat fragility and DNA damage. In Huntington’s disease, the single-stranded CTG DNA exposed within pathogenic CAG/CTG repeat expansion regions is prone to forming R-loops-dependent hairpin structures. These structures serve as substrates for the cytidine deaminase APOBEC3A (A3A), leading to cytidine deamination, which subsequently induces DNA double-strand breaks and genome instability [66]. Here, the dominant phenotype relates to repeat fragility rather than stable heterochromatin silencing.ALS/FTD highlights locus specificity and non-uniform causality. In C9ORF72 HRE-associated ALS/FTD models, promoter hypermethylation together with increased H3K9me3 is described as a principal mechanism of transcriptional silencing, and importantly this epigenetic perturbation can occur largely independent of R-loop accumulation at this locus [67].

Impaired R-loop processing and cellular vulnerability. Dysfunction of key R-loops metabolic regulators exacerbates neuronal injury. For instance, loss-of-function mutations in the critical R-loops helicase SETX, such as those associated with ataxia-telangiectasia-like disorder type 2 (ATLD2), impair its ability to form stable complexes with the RNA exosome core component Rrp45. This leads to diminished R-loops clearance and defective DNA damage repair, thereby accelerating motor neuron degeneration [68]. Similarly, the loss of ADAR1 abolishes its double-stranded RNA-binding capacity and adenosine-to-inosine RNA editing function, rendering unedited nascent RNA more prone to forming stable R-loops with DNA templates or impeding the clearance of pre-existing R-loops. This results in R-loops accumulation, hyperactivation of the ATR signalling pathway, and exacerbation of neuroinflammatory responses [69,70]. Such perturbations may be particularly deleterious in long-lived, transcriptionally active neural cells with limited capacity to dilute DNA lesions through cell division. Overall, neurodegenerative phenotypes appear to arise through multiple, partially overlapping routes – repeat-associated chromatin repression, repeat fragility/DSBs, and impaired R-loop clearance – rather than a single unifying R-loop mechanism.

#### Cancer: heterogeneous reciprocal interactions of R-loops and epigenetic networks in genome instability and tumor evolution

In cancer, altered R-loop homeostasis is frequently observed and can intersect with epigenetic dysregulation and genome instability; however, its contribution is highly context-dependent, varying with tumour type, oncogenic transcription programmes, replication stress, and the integrity of R-loops processing pathways [71].

RNA modification – dependent tuning of R-loops and repair outcomes. RNA epigenetic marks associated with R-loops can modulate hybrid stability and influence downstream chromatin or DNA repair responses. For example, METTL3-mediated m^6^A on R-loop – associated RNAs can be recognized by YTHDC1 and, in certain contexts such as telomere maintenance, is linked to stabilization of R-loop structures and homologous recombination repair [72]. On the other hand, m^5^C modification mediated by methyltransferase TRDMT1 enhances the affinity of recombination protein RAD52 for DNA-RNA hybrids, thereby promoting homologous recombination repair of DNA damage [37]. These studies indicate that R-loops can participate in pro-survival repair pathways in specific tumour settings rather than serving universally as damaging lesions.Promoter-localized epigenetic reprogramming is locus specific. At selected CpG-island promoters, R-loops can serve as platforms for recruitment of demethylation machinery, such as the GADD45A – TET1 axis at the tumour suppressor TCF21 [23]or GADD45A – TET3 recruitment by DSP-AS1-associated R-loops [22,24], leading to local 5hmC accumulation and transcriptional activation. The occurrence and impact of these mechanisms likely depend on promoter chromatin state and availability of the relevant effectors in a given tumor.Chromatin modifier dysfunction can also act upstream of R-loop accumulation. Epigenetic alterations that weaken chromatin compaction may predispose to R-loop formation by intensifying transcription – replication conflicts. For instance, loss of SUV420H2 reduces H4K20me3 and heterochromatin compaction in right-sided colon cancer [73]. Deficiency in PRC1-mediated H2AK119ub1 similarly compromises chromatin stability and is associated with increased RNA:DNA hybrids, replication fork stalling, and DNA breaks [50,51]. Conversely, stabilized hybrids can also function downstream to recruit PRC2 effectors (e.g., EZH2) and promote H3K27me3 deposition at selected tumour suppressor loci (for example, in NSUN2–m5C – linked contexts), highlighting bidirectional and locus-dependent Polycomb – R-loops coupling. This impairs chromatin stability, exacerbates transcription-replication conflicts, promotes abnormal R-loops accumulation, and ultimately impedes replication fork progression while inducing DNA breaks.

Thus, describing R-loops as a single unifying oncogenic axis may be overly reductive; in different tumours they may act as drivers, modifiers, vulnerabilities, or compensatory adaptations within broader epigenetic and replication-stress landscapes.Notably, under normal physiological conditions, R-loops formation and clearance are precisely regulated and play essential roles [74].

#### Metabolic disorders and developmental abnormalities: tissue- and stage-dependent consequences of R-loop dysregulation

Evidence linking R-loops to metabolic and developmental phenotypes is currently derived from a limited set of models and points to strong context specificity. In metabolic disease models, R-loops accumulation often appears secondary to perturbations in RNA processing or organelle homeostasis, whereas in development, R-loops can act as regulatory intermediates shaping epigenetic transitions.

Metabolic context. Loss of the splicing factor SRSF1 in a non-alcoholic steatohepatitis (NASH) model induces abnormal accumulation of R-loops in hepatocytes. The resulting DNA double-strand breaks, marked by γH2AX, significantly suppress the expression of key lipid metabolism genes such as PPARA and CPT1A, thereby promoting lipid accumulation and inflammatory cascade responses [75]. Separately, impaired p53-mediated degradation of RNA – DNA hybrids within the mitochondrial matrix induces mtDNA mutations and oxidative phosphorylation defects [76], highlighting an organelle-specific context distinct from nuclear chromatin-based mechanisms.Developmental context. In embryonic stem cells, ZFP281 binds R-loops and recruits DNMT3/TET1, influencing the balance of 5mC and 5hmC and thereby affecting pluripotency transitions and differentiation [77]. During retinal development, NRL-associated R-loops at long neuronal genes correlate with redistribution of H3K4me3/H3K27me3 and remodelling of 3D chromatin organization to regulate photoreceptor gene programmes [78]. In these settings, R-loops are intertwined with developmental epigenetic programming, and pathological outcomes likely arise when their dynamics are mis-timed or mis-localized.Overall, rather than representing a uniform ‘core pathological axis’ across organs, R-loop – epigenetic interactions in metabolic and developmental disorders appear to reflect tissue- and stage-dependent mechanisms, with distinct upstream perturbations and downstream chromatin or mitochondrial outcomes. For a consolidated overview of the specific mechanisms discussed, please refer to Table 1.Note: Listed leverage points reflect mechanisms interrogated in experimental systems. They are provided to illustrate hypothesis-generating directions and are not intended as near-term translational or clinical recommendations.

**Table 1.** Pathogenic mechanisms of R-loops – Epigenetic interplay in human.

Disease Category	Core Molecular Mechanism	Key Epigenetic Modification	Major Pathological Consequence	Representative Targets/Pathways	References
Neurodegenerative Diseases	R-loop recruitment of G9a by repetitive sequences	Aberrant H3K9me2 deposition	FXN gene silencing	G9a inhibitors	[63]
	SETX mutation causing defective R-loop clearance	R-loop accumulation	Failure of DNA damage repair	RNA exosome activators	[68]
	ADAR1 deficiency stabilizing R-loops	Loss of A-to-I editing	ATR overactivation, Neuroinflammation	ATR inhibitors	[69,70]
	SVA retrotransposon–embedded repeat RNA drives R-loop formation	Dysregulated ZNF91/DNMT1-mediated SVA silencing	Impaired neuronal activity, Apoptosis	ASO targeting repeat RNA	[65]
Cancer	METTL3-mediated m^6^ A modification stabilizing R-loop mRNA	m^6^ A modification of mRNA	Enhanced telomere homologous recombination	METTL3 inhibitors	[2,72]
	R-loops recruitment of the GADD45A-TET1 complex	TET1-mediated DNA demethylation	Tumor suppressor gene activation	GADD45A antagonists	[2,23]
	AND-1 regulates R-loops dynamics	Phosphorylation of AND-1 at T826	Endocrine resistance	AND-1	[79]
	Dysfunctional PRC1 complex	H2A ubiquitination imbalance	Replication fork stalling, DNA breaks	SIRT1/BMI1 modulators	[5,80]
Metabolic/ Developmental Diseases	Hepatic SRSF1 deficiency causing R-loop accumulation	p53-dependent DNA damage signaling	PPARA/CPT1A suppression, NASH inflammation	SRSF1 agonists	[75]
	ZFP281 deficiency in embryonic stem cells	DNMT3A/TET1 dynamic imbalance	Pluripotency transition blockade	ZFP281 gene therapy	[77]

RQ3: R-Loops Epigenetic Regulation Dynamic Profiling Technological Framework

Elucidating R-loops interactions with epigenetic regulation requires a multidimensional framework across four key domains: dynamic detection, epigenetic profiling, functional validation, and multi-omics integration. R-loops detection primarily uses S9.6-based DRIP-seq, validated by RNase H digestion, Dot-blot, and IF, with advanced methods like ULI-ssDRIP-seq and MapR improving low-input and in vivo tracking [81]. Epigenetic profiling includes DNA methylation/hydroxymethylation via WGBS, RRBS, methylation arrays, and hMeDIP; RNA modifications through MeRIP-seq, SID-UPLC-MS/MS, and miCLIP-seq; and histone marks via ChIP-seq and CUT&Tag. Functional validation employs CRISPR-Cas9 with siRNA/shRNA for gene modulation, Co-IP/RIP/PLA for interactions, and comet assays/γH_2_AX IF/nuclear run-on for impacts on DNA damage, replication, and transcription. Integrative analysis correlates DRIP-seq, RNA-seq, and ChIP-seq data using tools like R-loopDB and SRAMP to model networks [82]. Spatial heterogeneity is resolved with 3D-STORM imaging and single-cell sequencing. Overall, technologies evolve from static, bulk-level to dynamic, in situ, single-cell resolution, integrating with disease models to reveal R-loops roles in homeostasis and pathogenesis. (For detailed technology summaries, refer to the accompanying Table 2.)

**Table 2.** Research Methodology framework for R-Loops-mediated epigenetic regulation.

Technical Dimension	Core Methods	Technical Principles & Characteristics	References
1. R-loops Spatiotemporal Detection	S9.6 Antibody-based Methods:DRIP-seq, ssDRIP-seq, Dot-blot, Immunofluorescence (IF)	Specific recognition of RNA:DNA hybrids Genome-wide mapping	[19,22,83]
	Enzymatic Tracing:MapR, ULI-ssDRIP-seq, enDR3	MapR:RNase H1–micrococcal nuclease fusion proteinULI-ssDRIP-seq:Quantitative analysis with single-strand resolution	[27,81,84,85]
2. Epigenetic Modifications Analysis	DNA Modifications:WGBS/RRBS, hMeDIP	Genome-wide profiling of 5mC/5hmC Hydroxymethylation dynamics capture	[26,86]
	RNA Modifications:m^6^ A/m^5^ C MeRIP-seq, SID-UPLC-MS/MS	RNA methylation enrichment sequencing Mass spectrometry-based modification abundance quantification	[30,83,87]
	Histone Modifications:ChIP-seq, CUT&Tag	Genomic localization of marks (e.g., H3K4me3, H3K27ac)High-sensitivity detection with low background noise	[88,89]
3. Functional Mechanism Validation	Genetic Manipulation:CRISPR-Cas9 editing, siRNA/shRNA	Targeted modulation (e.g., RNase H1, TET2) Rapid loss-of-function validation	[30,90]
	Molecular Interactions:RIP/Co-IP, PLA	Protein–RNA hybrid interaction detection < 40 nm spatial proximity verification	[31,72,91]
	Pathological Models:Xenografts, Genetically engineered animals	Phenotypic correlations (e.g., DNA damage from R-loop dysregulation, developmental delays)	[22,44,85]
4. Integrated Analysis Platforms	Multi-omics Integration:DRIP-seq + RNA-seq + ChIP-seq	Construction of R-loop distribution, transcriptional activity, and epigenetic modification networks	[82,86,92]
	Advanced Technologies:3D-STORM imaging, Single-cell sequencing	Nanoscale spatial resolution Cellular heterogeneity assessment	[85,92]

## Discussion

R-loops have evolved from transcriptional byproducts into recurrent molecular intermediates at the interface of transcription, chromatin regulation, and genome maintenance [93]. Although the association between R-loops and epigenetic dysregulation in neurodegenerative diseases, cancer, metabolic disorders, and other diseases has become increasingly evident, translating these mechanisms into effective clinical interventions remains a significant challenge.

The core translational challenges converge on three dimensions: targeting specificity, delivery efficiency, and dynamic monitoring. The foremost challenge lies in the precise discrimination between pathogenic R-loops and physiologically functional R-loops, which necessitates the establishment of a more refined functional atlas of R-loops. Particularly prominent in the field of neurological disorders is the delivery barrier, where there is an urgent need to overcome the limitations imposed by the blood-brain barrier (BBB) on the delivery of candidate molecules currently explored in preclinical settings (e.g., antisense oligonucleotides targeting repeat RNAs, or small-molecule modulators of RNA-modifying enzymes such as METTL3) [65]. Importantly, these approaches are not R-loop-specific and may have broad, pleiotropic effects. This includes global RNase H perturbations, which can erase both pathogenic and physiological hybrids and therefore require catalytic-dead controls and orthogonal validation to support causal attribution. The bottleneck in dynamic monitoring lies in the lack of real-time, non-invasive techniques for assessing in vivo R-loops dynamics and their epigenetic effects, which severely impedes the optimization of therapeutic strategies. Current technical systems also exhibit limitations: traditional S9.6 antibodies carry the risk of cross-reactivity with non-target nucleic acids such as double-stranded RNA [94], while the highly dynamic nature of R-loops during developmental stages or cell cycle progression further complicates their resolution. To mitigate S9.6-related specificity concerns, newer enzyme-driven, antibody-independent mapping strategies (e.g., enDR3) have been developed for genome-wide R-loop profiling across in vivo and ex vivo contexts [81]. While novel strand-specific ssDRIP-seq approaches allow monitoring of R-loops dynamics in vertebrate embryogenesis [85,95], and stable isotope dilution coupled with ultrahigh-performance liquid chromatography-tandem mass spectrometry, abbreviated as SID-UHPLC-MS/MS, provides precise quantification of RNA modifications embedded within R-loops, including N6-methyladenosine and 5-methylcytidine [87], these techniques remain inadequate for real-time clinical monitoring applications. For mechanistic stratification in disease samples, one research-facing approach is to combine RNase H – controlled S9.6 readouts (and where feasible antibody-independent mapping) with chromatin-state marks aligned to the inferred pathway (e.g., H3K9me2 for G9a/GLP-driven repeat silencing versus H2AK119ub1/H3K27me3 for Polycomb-linked contexts), together with downstream stress markers (e.g., γH2AX/53BP1 or replication-stress signalling in proliferative tumours).A deeper challenge stems from the complexity of regulatory networks. The redundancy between R-loops and epigenetic modification networks means that interventions targeting single factors (e.g., RNA- or histone-modifying enzymes) may trigger compensatory responses, particularly given the pleiotropic roles of these regulators [96,97]. Therefore, any future therapeutic strategies will likely require a clearer definition of disease-context dependencies and careful validation of whether phenotypes are mediated through R-loops versus broader transcriptional effects. For example, AND-1 has been proposed as a regulator of R-loop dynamics in endocrine resistance models [79]; however, translation of such observations will depend on robust, R-loop-specific causal validation and clinically feasible assays.

Collectively, R-loops may represent a recurrent interface linking DNA methylation, RNA chemistry, histone marks, and 3D genome features in a context- and locus-dependent manner. Reciprocal influences and feedback-like patterns have been proposed in selected systems, but current evidence does not yet support a single generalizable regulatory architecture operating uniformly across cell types and diseases.

Dysregulation may contribute to self-reinforcing loops between epigenetic alterations, DNA damage, and impaired resolution capacity; however, the upstream triggers, chromatin contexts, and cell-type vulnerability that shape these feedbacks differ substantially across neurodegeneration, cancer, and developmental/metabolic disorders. Therefore, R-loops may represent a recurrent interface between transcription, chromatin, and genome maintenance rather than a single unifying pathogenic axis.Looking ahead, resolving these translational impasses demands multidisciplinary convergence. Advancing technologies with higher spatiotemporal resolution – such as refined live-cell imaging, single-cell multi-omics, and super-resolution microscopy – is crucial for real-time dissection of R-loop formation, maintenance, resolution, and their epigenetic sequelae. Concurrently, leveraging computational biology and artificial intelligence to integrate expansive datasets (R-loops mapping, epigenomic profiles, transcriptomes, proteomes and 3D architectures) may enable quantitative multilayer network models to define disease-relevant modules and prioritize candidate molecular interfaces for further mechanistic investigation [82]. Such predictions should be interpreted cautiously and ideally supported by benchmarking across platforms and orthogonal perturbation – rescue validation, particularly for 3D contact-map comparisons where analytical choices can yield discordant results. Critically, the role of R-loops in acute pathological conditions remains unclear, particularly in acute processes such as cardiac arrest and ischaemia – reperfusion injury, where their potential involvement in epigenetic regulation has yet to be thoroughly investigated and warrants further exploration. Furthermore, it remains unknown whether and how environmental factors such as temperature changes and hypoxia regulate the dynamic formation and function of R-loops. Previous studies have revealed abundant epigenetic regulatory mechanisms in ischaemia – reperfusion injury and therapeutic hypothermia [98,99]. Elucidating R-loop functions in these acute and environmentally modulated contexts is essential to comprehend the full spectrum of their epigenetic regulation and may uncover context-specific mechanistic entry points or biomarker candidates that could, in the longer term, be explored for therapeutic relevance after rigorous validation.

## Supplementary Material

Supplemental Material

## Data Availability

Data sharing is not applicable to this article as no data were created in this study.
