# Supplementary material for: The role of R-loops-mediated epigenetic regulation in genome stability maintenance and disease pathogenesis: a systematic review
Source: Epigenetics. 2026 Apr 13;21(1):2653959. doi: 10.1080/15592294.2026.2653959 (PMC13078211; doi:10.1080/15592294.2026.2653959)
Supplement: Supplemental Material [file KEPI_A_2653959_SM9311.zip › Appendix - Search Strategy.docx]

("R-loop" OR "DNA:RNA hybrid" OR "R loops" OR "RNA-DNA hybrid") AND

(epigenetic OR "epigenetic regulation" OR "epigenetic modification" OR "epigenetic mechanisms" OR "DNA methylation" OR "DNA hydroxymethylation" OR "DNA demethylation" OR "DNA base modification" OR "DNA cross-linking" OR"histone modification" OR "histone acetylation" OR "histone methylation" OR "histone phosphorylation" OR"RNA modification" OR "RNA methylation" OR "RNA demethylation" OR "m6A" OR "N6-methyladenosine" OR "RNA editing" OR "pseudouridylation" OR "RNA splicing" OR"non-coding RNA" OR ncRNA OR lncRNA OR microRNA OR miRNA OR siRNA OR"post-translational modification" OR "protein modification" OR phosphorylation OR acetylation OR methylation OR ubiquitination OR SUMOylation OR glycosylation OR lipidation OR sulfation OR pantothenylation OR"chromatin remodeling" OR "chromatin structure" OR "chromatin folding" OR "chromatin architecture" OR"three-dimensional genome" OR "3D genome" OR "topologically associating domains" OR TADs OR CTCF OR cohesin OR"genomic region interactions" OR "chromosomal loop" OR "chromosome conformation capture" OR Hi-C OR "nuclear gene organization")
